# Supplementary material for: Acid Soil Improvement Enhances Disease Tolerance in Citrus Infected by Candidatus Liberibacter asiaticus
Source: Int J Mol Sci. 2020 May 20;21(10):3614. doi: 10.3390/ijms21103614 (PMC7279377; doi:10.3390/ijms21103614)
Supplement: Supplementary file 1 [file ijms-21-03614-s001.pdf]

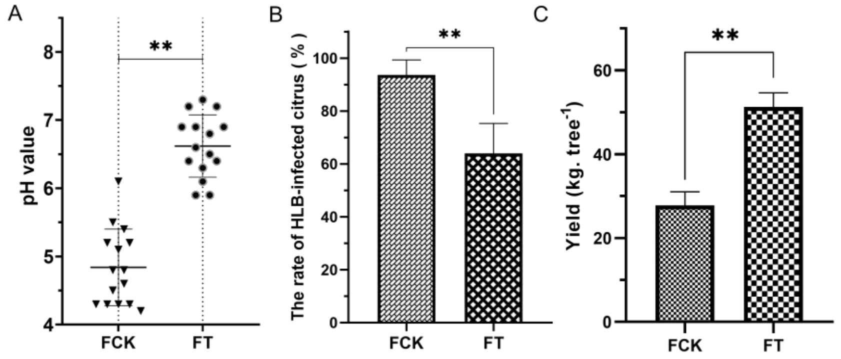

Figure S1. Investigation of soil pH values and production index in the field trial in 2018. (**A**) Soil pH value of the FCK and FT in November 2018. (**B**) The new infections rate of HLB disease in the FCK and FT in November 2018. Data represent the mean ± SD of three independent biological replicates with 100 plants per replicate. (**C**) Yield of HLB-infected citrus trees cultivated under two different soil acidity conditions in November 2018. Data represent the mean ± SD (n = 20). FCK, control grove in field trial; FT, treatment grove in field trial. Asterisks indicate a significant difference according to the Student's t-test: $**p < 0.01$.

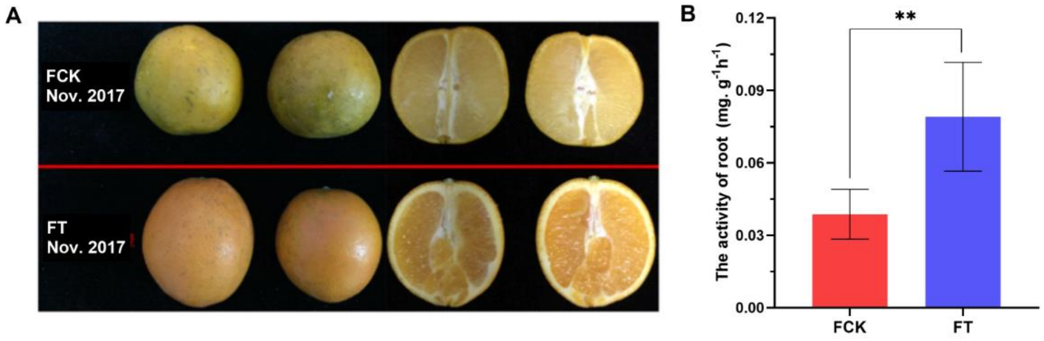

Figure S2. Effect of acid soil improvement on fruit morphology and root metabolic activity of citrus trees with Huanglongbing (HLB) infection in the field trial. (**A**) Appearance and peel color of HLB-infected citrus fruits, which are harvested from the control grove (top) and the treatment grove (bottom). (**B**) The root activity of HLB-infected citrus seedling samples in the FCK and FT was measured by TTC method at the fifth month after repotting. Data represent the mean ± SD of four independent biological replicates. FCK, control group in greenhouse trial; FT, treatment group in greenhouse trial. Asterisks indicate a significant difference according to the Student's t-test: ** $p < 0.01$.

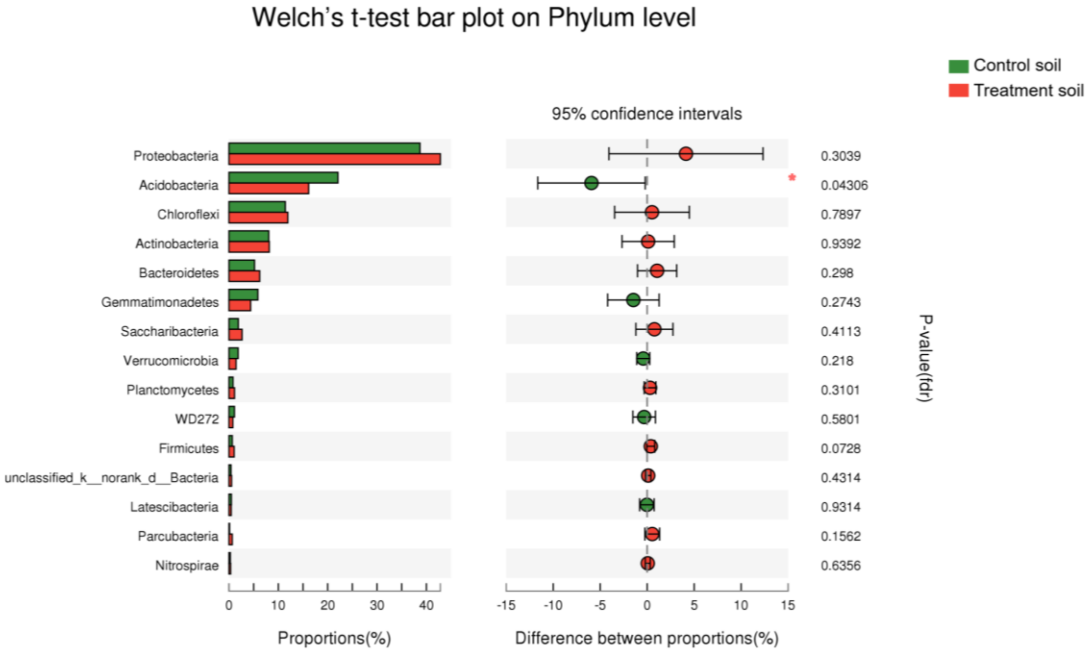

Figure S3. The changes of bacterial communities at the Phylum level in rhizosphere soil of HLB-infected citrus under the different soil conditions. Asterisks indicate a significant difference : $*p < 0.05$.

Table S1. Information of primers used in this study.

Primer name	Forward Sequence(5'-3')	Reserve Sequence(5'-3')
CsPR1	TCTTGTGCATTCGGGAGGAC	CACCCAATGCGAACCGAATT
CsPR5	TCTCATTGGGCATTGTGGCT	ATCACCGGTTGTGCATTTGC
CsNPR1	GCTCGCATGTTGCTTGTAGG	GAGCCACCAACTCTGGAACC
CsALD1	GGCATTGAAGGAGACGAA	TCCAAGAAGCATCTGTAGAC
CsWRKY1	ACTTGCTGCCATCGGATCAA	TGCCCATACTTTCGCCAGTT
CsWRKY2	GGTGAGAAAGCACGTGGAGA	TTGCCATGCTGTTGTGAAGC
CsZIP1	GAAGCGGAGGATAACG	CCGATTCACCAGCATAT
CsVIT1	ATAGAGGCTGCTGAGTGTGC	AGCCAATGTTTTGGGTTCCTC
csAmtB	TCTGGAGTTGCTGGTTAC	CCCGCTAACATTAGAAGGA
CsBOR1	ATGGTGTGCCACTGATGGTT	ACTGAAGAGGCGCCTAGGAA
CsBOR1	ATGGTGTGCCACTGATGGTT	ACTGAAGAGGCGCCTAGGAA
CsGAPDH	GGAAGGTCAAGATCGGAATCA	CGCCCTCTGCAAGATGACTCT

Table S2. Differentially expressed proteins of HLB-infected citrus midribs from different soil pH conditions.

Table S2 All the differentially expressed proteins of HLB-infected citrus midribs from different soil pH conditions.

A total of 119 differentially expressed proteins at a cutoff value of >|±1.5|-fold (p value <0.005)

Identified Proteins	UniProtKB Entry	Mann-Whitney Test (p-value) Benjamini-Hochberg (p < 0.02450)	Log2 Fold Change by Category	Fold Change by Category	Regulated Type
Uncharacterized protein OS=Citrus sinensis GN=CISIN_1g031515mg PE=3 SV=1	A0A067G1V8	0.0039	-1.81	-3.51	Down
Uncharacterized protein OS=Citrus sinensis GN=CISIN_1g026695mg PE=3 SV=1	A0A067EBG5	0.0001	-1.61	-3.05	Down
Uncharacterized protein (Fragment) OS=Citrus sinensis GN=CISIN_1g036405mg PE=3 SV=1	A0A067D515	0.0001	-1.6	-3.03	Down
Uncharacterized protein OS=Citrus sinensis GN=CISIN_1g029999mg PE=4 SV=1	A0A067EZU9	0.0001	-1.52	-2.87	Down
Uncharacterized protein OS=Citrus sinensis GN=CISIN_1g043296mg PE=3 SV=1	A0A067EJK1	0.0001	-1.24	-2.36	Down
Uncharacterized protein OS=Citrus sinensis GN=CISIN_1g043288mg PE=3 SV=1	A0A067DHX9	0.0013	-1.17	-2.25	Down
Uncharacterized protein OS=Citrus sinensis GN=CISIN_1g011914mg PE=4 SV=1	A0A067EBB3	0.0001	-1.13	-2.19	Down
Uncharacterized protein OS=Citrus sinensis GN=CISIN_1g028798mg PE=3 SV=1	A0A067DV78	0.0001	-1.11	-2.16	Down
Uncharacterized protein OS=Citrus sinensis GN=CISIN_1g0264162mg PE=4 SV=1	A0A067G8Q7	0.0001	-1.11	-2.16	Down
Uncharacterized protein OS=Citrus sinensis GN=CISIN_1g031533mg PE=3 SV=1	A0A067DAV6	0.0017	-1.08	-2.11	Down
Uncharacterized protein OS=Citrus sinensis GN=CISIN_1g005321mg PE=3 SV=1	A0A067G9H4	0.0001	-1.06	-2.08	Down

Uncharacterized protein OS=Citrus sinensis GN=CISIN_1g027026mg PE=3 SV=1	A0A067DFS4	0.0001	-1.04	-2.06	Down
Uncharacterized protein (Fragment) OS=Citrus sinensis GN=CISIN_1g0223951mg PE=4 SV=1	A0A067E2W1	0.009	-1.02	-2.03	Down
Uncharacterized protein OS=Citrus sinensis GN=CISIN_1g006194mg PE=3 SV=1	A0A067EQV8	0.0001	-1.01	-2.01	Down
Uncharacterized protein OS=Citrus sinensis GN=CISIN_1g0316021mg PE=3 SV=1	A0A067E4D3	0.0001	-0.99	-1.99	Down
Uncharacterized protein OS=Citrus sinensis GN=CISIN_1g031365mg PE=3 SV=1	A0A067FU45	0.0001	-0.96	-1.95	Down
Uncharacterized protein OS=Citrus sinensis GN=CISIN_1g031218mg PE=3 SV=1	A0A067FTG1	0.0001	-0.92	-1.89	Down
Uncharacterized protein (Fragment) OS=Citrus sinensis GN=CISIN_1g0459451mg PE=4 SV=1	A0A067DS64	0.0017	-0.9	-1.87	Down
Uncharacterized protein OS=Citrus sinensis GN=CISIN_1g033674mg PE=4 SV=1	A0A067DM63	0.0001	-0.86	-1.82	Down
Putative gibberellin-regulated protein OS=Citrus sinensis GN=CISIN_1g034269mg PE=4 SV=1	A0A067DAR4	0.0017	-0.86	-1.82	Down
Uncharacterized protein OS=Citrus sinensis GN=CISIN_1g029075mg PE=4 SV=1	A0A067E2X5	0.0001	-0.83	-1.78	Down
Uncharacterized protein OS=Citrus sinensis GN=CISIN_1g026736mg PE=3 SV=1	A0A067H290	0.0001	-0.82	-1.77	Down
Uncharacterized protein OS=Citrus sinensis GN=CISIN_1g031937mg PE=3 SV=1	A0A067FH96	0.0001	-0.82	-1.77	Down
Uncharacterized protein OS=Citrus sinensis GN=CISIN_1g025588mg PE=3 SV=1	A0A067EJB8	0.0001	-0.81	-1.75	Down
Uncharacterized protein OS=Citrus sinensis GN=CISIN_1g031500mg PE=4 SV=1	A0A067FUA9	0.0001	-0.8	-1.74	Down
Uncharacterized protein OS=Citrus sinensis GN=CISIN_1g025388mg PE=3 SV=1	A0A067GEG2	0.0001	-0.74	-1.67	Down
Uncharacterized protein OS=Citrus sinensis GN=CISIN_1g023621mg PE=4 SV=1	A0A067F9Z8	0.0001	-0.74	-1.67	Down
Uncharacterized protein OS=Citrus sinensis GN=CISIN_1g032466mg PE=4 SV=1	A0A067DPR4	0.0001	-0.73	-1.66	Down
Uncharacterized protein OS=Citrus sinensis GN=CISIN_1g048141mg PE=4 SV=1	A0A067DWN3	0.009	-0.73	-1.66	Down
Amine oxidase OS=Citrus sinensis GN=CISIN_1g006085mg PE=3 SV=1	A0A067GGT5	0.0001	-0.72	-1.65	Down
Uncharacterized protein OS=Citrus sinensis GN=CISIN_1g021718mg PE=3 SV=1	A0A067FT01	0.0001	-0.71	-1.64	Down

Uncharacterized protein OS=Citrus sinensis GN=CISIN_1g005260mg PE=4 SV=1	A0A067FGT2	0.0001	-0.71	-1.64	Down
Uncharacterized protein OS=Citrus sinensis GN=CISIN_1g028468mg PE=3 SV=1	A0A067D698	0.00016	-0.71	-1.64	Down
Uncharacterized protein OS=Citrus sinensis GN=CISIN_1g027048mg PE=3 SV=1	A0A067D7K7	0.0001	-0.7	-1.62	Down
Uncharacterized protein OS=Citrus sinensis GN=CISIN_1g017915mg PE=4 SV=1	A0A067EPI2	0.0001	-0.7	-1.62	Down
Uncharacterized protein OS=Citrus sinensis GN=CISIN_1g009278mg PE=4 SV=1	A0A067E3F4	0.0001	-0.69	-1.61	Down
Uncharacterized protein OS=Citrus sinensis GN=CISIN_1g046258mg PE=3 SV=1	A0A067EHH8	0.0001	-0.68	-1.6	Down
Uncharacterized protein OS=Citrus sinensis GN=CISIN_1g031640mg PE=3 SV=1	A0A067D4J5	0.0001	-0.68	-1.6	Down
Uncharacterized protein OS=Citrus sinensis GN=CISIN_1g019870mg PE=4 SV=1	A0A067GIE1	0.0001	-0.67	-1.59	Down
Carbonic anhydrase OS=Citrus sinensis GN=CISIN_1g019915mg PE=3 SV=1	A0A067FFU3	0.0017	-0.67	-1.59	Down
Uncharacterized protein OS=Citrus sinensis GN=CISIN_1g047658mg PE=4 SV=1	A0A067FZ61	0.0001	-0.66	-1.58	Down
Uncharacterized protein OS=Citrus sinensis GN=CISIN_1g025070mg PE=4 SV=1	A0A067H6Z0	0.0001	-0.66	-1.58	Down
Carbonic anhydrase OS=Citrus sinensis GN=CISIN_1g019915mg PE=3 SV=1	A0A067FRU7	0.009	-0.66	-1.58	Down
Uncharacterized protein OS=Citrus sinensis GN=CISIN_1g027901mg PE=4 SV=1	A0A067E2C3	0.0001	-0.64	-1.56	Down
Uncharacterized protein OS=Citrus sinensis GN=CISIN_1g033992mg PE=4 SV=1	A0A067FPH1	0.00035	-0.64	-1.56	Down
Uncharacterized protein OS=Citrus sinensis GN=CISIN_1g030557mg PE=4 SV=1	A0A067DSE9	0.009	-0.64	-1.56	Down
Uncharacterized protein OS=Citrus sinensis GN=CISIN_1g038064mg PE=4 SV=1	A0A067DAL3	0.00035	-0.63	-1.55	Down
Uncharacterized protein (Fragment) OS=Citrus sinensis GN=CISIN_1g046801mg PE=4 SV=1	A0A067DD81	0.0001	-0.62	-1.54	Down
Uncharacterized protein OS=Citrus sinensis GN=CISIN_1g005707mg PE=4 SV=1	A0A067FGH8	0.0001	-0.62	-1.54	Down
Uncharacterized protein OS=Citrus sinensis GN=CISIN_1g002012mg PE=3 SV=1	A0A067FW96	0.0001	-0.61	-1.53	Down
PS1 reaction center subunit III OS=Citrus sinensis GN=psaE PE=2 SV=1	A8C1A6	0.0001	-0.61	-1.53	Down
Uncharacterized protein OS=Citrus sinensis GN=CISIN_1g022816mg PE=4 SV=1	A0A067E0C7	0.0001	-0.61	-1.53	Down
Uncharacterized protein OS=Citrus sinensis GN=CISIN_1g022296mg PE=4 SV=1	A0A067GJW8	0.0001	-0.61	-1.53	Down

Uncharacterized protein OS=Citrus sinensis GN=CISIN_1g021202mg PE=4 SV=1	A0A067GL32	0.0001	-0.61	-1.53	Down
Uncharacterized protein OS=Citrus sinensis GN=CISIN_1g023679mg PE=4 SV=1	A0A067H729	0.009	-0.61	-1.53	Down
Uncharacterized protein OS=Citrus sinensis GN=CISIN_1g025092mg PE=4 SV=1	A0A067DPL0	0.0001	-0.6	-1.52	Down
Uncharacterized protein OS=Citrus sinensis GN=CISIN_1g012885mg PE=4 SV=1	A0A067E370	0.0001	-0.6	-1.52	Down
Uncharacterized protein OS=Citrus sinensis GN=CISIN_1g005815mg PE=3 SV=1	A0A067GB16	0.0001	-0.6	-1.52	Down
Uncharacterized protein OS=Citrus sinensis GN=CISIN_1g024728mg PE=4 SV=1	A0A067GWU3	0.0001	-0.6	-1.52	Down
Uncharacterized protein OS=Citrus sinensis GN=CISIN_1g002068mg PE=3 SV=1	A0A067GBF7	0.0001	-0.59	-1.51	Down
Uncharacterized protein OS=Citrus sinensis GN=CISIN_1g043550mg PE=4 SV=1	A0A067EDT6	0.0001	-0.59	-1.51	Down
Uncharacterized protein OS=Citrus sinensis GN=CISIN_1g031707mg PE=4 SV=1	A0A067GP16	0.0017	-0.59	-1.51	Down
Uncharacterized protein OS=Citrus sinensis GN=CISIN_1g010083mg PE=4 SV=1	A0A067F681	0.021	0.59	1.51	Up
Uncharacterized protein (Fragment) OS=Citrus sinensis GN=CISIN_1g036835mg PE=4 SV=1	A0A067DZ96	0.00078	0.58	1.51	Up
Uncharacterized protein OS=Citrus sinensis GN=CISIN_1g014537mg PE=3 SV=1	A0A067DIT7	0.0001	0.59	1.51	Up
Lipoxygenase OS=Citrus sinensis GN=CISIN_1g002672mg PE=3 SV=1	A0A067FFK4	0.0001	0.59	1.51	Up
Uncharacterized protein OS=Citrus sinensis GN=CISIN_1g012744mg PE=4 SV=1	A0A067GBM2	0.0001	0.59	1.51	Up
Beta_HSD domain-containing protein OS=Citrus sinensis GN=CISIN_1g0202661mg PE=4 SV=1	A0A067FCS1	0.0001	0.59	1.51	Up
Serine-type carboxypeptidase OS=Citrus sinensis GN=CISIN_1g031873mg PE=3 SV=1	A0A067D666	0.0001	0.59	1.51	Up
Methyltransf_2 domain-containing protein OS=Citrus sinensis GN=CISIN_1g048196mg PE=3 SV=1	A0A067DNI8	0.009	0.59	1.51	Up
Uncharacterized protein (Fragment) OS=Citrus sinensis GN=CISIN_1g0127112mg PE=4 SV=1	A0A067DPI6	0.00035	0.59	1.51	Up
Uncharacterized protein OS=Citrus sinensis GN=CISIN_1g014803mg PE=3 SV=1	A0A067ED11	0.0001	0.6	1.52	Up

Uncharacterized protein OS=Citrus sinensis GN=CISIN_1g008384mg PE=4 SV=1	A0A067ECS9	0.0001	0.6	1.52	Up
Uncharacterized protein OS=Citrus sinensis GN=CISIN_1g009399mg PE=4 SV=1	A0A067G6J5	0.0001	0.6	1.52	Up
Uncharacterized protein OS=Citrus sinensis GN=CISIN_1g005867mg PE=4 SV=1	A0A067H7I6	0.0001	0.6	1.52	Up
Serine/threonine-protein kinase like protein OS=Citrus sinensis GN=CISIN_1g001767mg PE=4 SV=1	A0A067DHQ0	0.0001	0.61	1.53	Up
Uncharacterized protein OS=Citrus sinensis GN=CISIN_1g024960mg PE=4 SV=1	A0A067DGD4	0.0001	0.61	1.53	Up
Uncharacterized protein (Fragment) OS=Citrus sinensis GN=CISIN_1g0099912mg PE=4 SV=1	A0A067E492	0.0001	0.62	1.54	Up
Carboxypeptidase OS=Citrus sinensis GN=CISIN_1g016137mg PE=3 SV=1	A0A067EBY4	0.009	0.62	1.54	Up
Uncharacterized protein (Fragment) OS=Citrus sinensis GN=CISIN_1g0063012mg PE=3 SV=1	A0A067F229	0.016	0.62	1.54	Up
Alpha-galactosidase (Fragment) OS=Citrus sinensis GN=CISIN_1g0388172mg PE=3 SV=1	A0A067ELD8	0.0001	0.63	1.55	Up
S-adenosylmethionine-dependent methyltransferases OS=Citrus sinensis GN=CISIN_1g0225151mg PE=3 SV=1	A0A067DIU3	0.0001	0.63	1.55	Up
Uncharacterized protein OS=Citrus sinensis GN=CISIN_1g020927mg PE=3 SV=1	A0A067EL94	0.021	0.63	1.55	Up
Uncharacterized protein (Fragment) OS=Citrus sinensis GN=CISIN_1g0274422mg PE=4 SV=1	A0A067GHE0	0.0001	0.64	1.56	Up
Uncharacterized protein OS=Citrus sinensis GN=CISIN_1g023045mg PE=4 SV=1	A0A067GE84	0.0027	0.64	1.56	Up
Uncharacterized protein OS=Citrus sinensis GN=CISIN_1g030668mg PE=4 SV=1	A0A067HGZ2	0.00035	0.65	1.57	Up
Uncharacterized protein OS=Citrus sinensis GN=CISIN_1g021920mg PE=4 SV=1	A0A067DFZ9	0.0001	0.66	1.58	Up
Uncharacterized protein OS=Citrus sinensis GN=CISIN_1g019168mg PE=4 SV=1	A0A067DWQ3	0.0001	0.66	1.58	Up
Uncharacterized protein OS=Citrus sinensis GN=CISIN_1g0095352mg PE=3 SV=1	A0A067G7A9	0.00021	0.66	1.58	Up

Uncharacterized protein OS=Citrus sinensis GN=CISIN_1g020633mg PE=4 SV=1	A0A067D7D1	0.0017	0.66	1.58	Up
Uncharacterized protein OS=Citrus sinensis GN=CISIN_1g0137801mg PE=4 SV=1	A0A067EYT5	0.0001	0.67	1.59	Up
Uncharacterized protein OS=Citrus sinensis GN=CISIN_1g037859mg PE=3 SV=1	A0A067GAK6	0.006	0.67	1.59	Up
Uncharacterized protein OS=Citrus sinensis GN=CISIN_1g034649mg PE=4 SV=1	A0A067FR03	0.00035	0.67	1.59	Up
Uncharacterized protein (Fragment) OS=Citrus sinensis GN=CISIN_1g0404312mg PE=4 SV=1	A0A067DD43	0.021	0.67	1.59	Up
SCP domain-containing protein (Fragment) OS=Citrus sinensis GN=CISIN_1g043403mg PE=3 SV=1	A0A067DC18	0.0001	0.68	1.6	Up
Uncharacterized protein (Fragment) OS=Citrus sinensis GN=CISIN_1g0308111mg PE=4 SV=1	A0A067GBI8	0.0039	0.68	1.6	Up
Uncharacterized protein (Fragment) OS=Citrus sinensis GN=CISIN_1g048392mg PE=4 SV=1	A0A067GIH9	0.009	0.68	1.6	Up
Glyceraldehyde-3-phosphate dehydrogenase OS=Citrus sinensis GN=CISIN_1g012940mg PE=3 SV=1	A0A067GZQ5	0.0001	0.69	1.61	Up
Alpha-amylase OS=Citrus sinensis GN=CISIN_1g014447mg PE=3 SV=1	A0A067GX71	0.0001	0.69	1.61	Up
Dirigent protein OS=Citrus sinensis GN=CISIN_1g029594mg PE=4 SV=1	A0A067F6J2	0.0001	0.7	1.62	Up
Uncharacterized protein (Fragment) OS=Citrus sinensis GN=CISIN_1g0476512mg PE=4 SV=1	A0A067F8S0	0.0001	0.72	1.65	Up
Uncharacterized protein OS=Citrus sinensis GN=CISIN_1g018914mg PE=4 SV=1	A0A067GHD8	0.009	0.72	1.65	Up
Uncharacterized protein (Fragment) OS=Citrus sinensis GN=CISIN_1g0356171mg PE=4 SV=1	A0A067EWM9	0.009	0.73	1.66	Up
Dirigent protein OS=Citrus sinensis GN=CISIN_1g029641mg PE=4 SV=1	A0A067FU00	0.00012	0.74	1.67	Up
Uncharacterized protein OS=Citrus sinensis GN=CISIN_1g020188mg PE=4 SV=1	A0A067DGV8	0.0001	0.76	1.69	Up

Uncharacterized protein OS=Citrus sinensis GN=CISIN_1g004301mg PE=4 SV=1	A0A067GJZ6	0.0001	0.76	1.69	Up
Serine-type carboxypeptidase OS=Citrus sinensis GN=CISIN_1g029289mg PE=3 SV=1	A0A067D7I5	0.00035	0.77	1.71	Up
Uncharacterized protein OS=Citrus sinensis GN=CISIN_1g031084mg PE=3 SV=1	A0A067E9I3	0.0001	0.78	1.72	Up
Dirigent protein OS=Citrus sinensis GN=CISIN_1g029746mg PE=4 SV=1	A0A067F9Y6	0.0039	0.78	1.72	Up
Uncharacterized protein OS=Citrus sinensis GN=CISIN_1g025959mg PE=4 SV=1	A0A067E2V5	0.0001	0.81	1.75	Up
Terpene synthase Terpene domain-containing protein OS=Citrus sinensis GN=CISIN_1g024780mg PE=3 SV=1	A0A067DU63	0.0011	0.86	1.82	Up
Uncharacterized protein (Fragment) OS=Citrus sinensis GN=CISIN_1g0092421mg PE=4 SV=1	A0A067GHZ7	0.0039	0.86	1.82	Up
Uncharacterized protein OS=Citrus sinensis GN=CISIN_1g030729mg PE=4 SV=1	A0A067EP14	0.0001	0.87	1.83	Up
O-methyltransferase OS=Citrus sinensis GN=CISIN_1g036188mg PE=3 SV=1	A0A067E1I5	0.00035	0.88	1.84	Up
Uncharacterized protein OS=Citrus sinensis GN=CISIN_1g025046mg PE=4 SV=1	A0A067E557	0.0001	0.89	1.85	Up
Uncharacterized protein OS=Citrus sinensis GN=CISIN_1g033108mg PE=4 SV=1	A0A067GVA6	0.0039	0.95	1.93	Up
Uncharacterized protein (Fragment) OS=Citrus sinensis GN=CISIN_1g0447211mg PE=4 SV=1	A0A067DQ49	0.0001	0.99	1.99	Up
SAM dependent carboxyl methyltransferase OS=Citrus sinensis GN=CISIN_1g017514mg PE=4 SV=1	A0A067FUD3	0.00078	1.34	2.53	Up
Pathogenesis-related thaumatin-like protein 5 OS=Citrus sinensis GN=CISIN_1g026001mg PE=4 SV=1	A0A067H9X4	0.0039	1.37	2.58	Up
